# A microbial-based cancer vaccine for induction of EGFRvIII-specific CD8+ T cells and anti-tumor immunity

**DOI:** 10.1371/journal.pone.0209153

**Published:** 2019-01-02

**Authors:** Lauren Zebertavage, Shelly Bambina, Jessica Shugart, Alejandro Alice, Kyra D. Zens, Peter Lauer, Bill Hanson, Michael J. Gough, Marka R. Crittenden, Keith S. Bahjat

**Affiliations:** 1 Earle A. Chiles Research Institute, Providence Portland Medical Center, Portland, OR, United States of America; 2 Oregon Health and Sciences University, Portland, OR, United States of America; 3 Aduro Biotech, Berkeley, CA, United States of America; 4 The Oregon Clinic, Portland, OR, United States of America; Monash University, Australia, AUSTRALIA

## Abstract

Dysregulated signaling via the epidermal growth factor receptor (EGFR)-family is believed to contribute to the progression of a diverse array of cancers. The most common variant of EGFR is EGFRvIII, which results from a consistent and tumor-specific in-frame deletion of exons 2–7 of the EGFR gene. This deletion generates a novel glycine at the junction and leads to constitutive ligand-independent activity. This junction forms a novel shared tumor neo-antigen with demonstrated immunogenicity in both mice and humans. A 21-amino acid peptide spanning the junctional region was selected, and then one or five copies of this 21-AA neo-peptide were incorporated into live-attenuated *Listeria monocytogenes*-based vaccine vector. These vaccine candidates demonstrated efficient secretion of the recombinant protein and potent induction of EGFRvIII-specific CD8+ T cells, which prevented growth of an EGFRvIII-expressing squamous cell carcinoma. These data demonstrate the potency of a novel cancer-specific vaccine candidate that can elicit EGFRvIII-specific cellular immunity, for the purpose of targeting EGFRvIII positive cancers that are resistant to conventional therapies.

## Introduction

The epidermal growth factor receptor (EGFR) is a receptor tyrosine kinase critical for cell growth and survival. Overexpression of EGFR is frequently associated with human cancers including breast cancer, non-small cell lung cancer, ovarian cancer and malignant glioma [1–3]. Approximately 40% of glioblastoma multiforme (GBM) patients have tumors that overexpress EGFR [2]. Of these cases, approximately 70% also express a mutant form of the EGFR [4]. The most common of these mutations is EGFR variant III (EGFRvIII), resulting from a deletion of 267 amino acids spanning exons 2–7 of the EGFR gene [5]. This alteration of the ligand-binding domain of EGFR alters the ability of the receptor to bind to its canonical ligands and produces low-level constitutive signaling activity [6]. Unlike wild-type (wt) EGFR, EGFRvIII can form both homodimers as well as heterodimers with wt-EGFR and Her2, thus, EGFRvIII signals may differ from those elicited by wt-EGFR [4]. These atypical signaling pairs could explain why tumors expressing EGFRvIII are unusually resistant to the effects of tyrosine kinase inhibitors [7] and anti-EGFR antibodies such as cetuximab [8]. In addition to promoting proliferation, EGFRvIII expression up-regulates the anti-apoptotic molecule Bcl-x_L_ and has been shown to mediate resistance to chemotherapeutic agents such as paclitaxel and cisplatin [9]. The presence of EGFRvIII also facilitates the STAT3-dependent induction of HIF-1α and promotes cell motility, invasion and metastasis [10]. Furthermore, expression of EGFRvIII by a subset of cells within a solid tumor can promote survival of EGFRvIII-negative cells via the IL-6/LIF/gp130-dependent induction of wt-EGFR [11]. These findings correlate with clinical data demonstrating that EGFRvIII expression in the presence of EGFR amplification is an indicator of a poor survival prognosis in GBM [12], and that EGFRvIII expression independently correlates with poor prognosis in patients with gross-total resection (>95%) surviving ≥1 year [13]. Therefore, EGFRvIII-expressing cells have a selective survival advantage over those expressing only wt-EGFR, and this advantage may become more pronounced after treatment.

Previous studies have shown that patients with EGFRvIII-expressing cancers have spontaneously developed humoral and cellular immune responses against EGFRvIII, suggesting that EGFRvIII serves as an immunogenic neo-antigen [14]. A 13 amino acid peptide from the unique splice junction of EGFRvIII (LEEKKGNYVVTDH), referred to as PEPvIII, has been used to vaccinate humans with EGFRvIII-expressing GBM. A phase 2 clinical trial of rindopepimut, a peptide vaccine containing PEPvIII conjugated to the carrier protein keyhole limpet hemocyanin (KLH), administered with adjuvant GM-CSF was performed in newly diagnosed GBM patients treated by gross total resection, radiation and temozolomide who had no radiographic evidence of progression. Humoral immune responses to EGFRvIII were observed in 6 of 14 immunized patients, while 3 of 17 showed a positive delayed type hypersensitivity (DTH) response. The median overall survival for patients treated with vaccine in combination with temozolomide was 26.0 months from the time of histologic diagnosis, versus 15.0 months for a matched cohort receiving only temozolomide [15]. However, a follow-up randomized, double-blind phase 3 trial of 745 patients (405 with minimal residual disease and 338 with significant residual disease, following maximal surgical resection and chemoradiation) treated with rindopepimut and temozolomide found no significant difference between patients receiving the investigational vaccine and patients treated with KLH and temozolomide. Intriguingly, in the small number of post-treatment samples obtained, EGFRvIII was lost equivalently in both the rindopepimut and control-treated patients [16]. Taken together, these results support the utility of EGFRvIII as an immunotherapeutic target, but suggest a vaccine with improved potency relative to PepvIII-KLH may achieve the desired outcome.

Intracellular microbes elicit a robust CD8^+^ T cell response in immunocompetent hosts, a response necessary to kill infected cells and prevent microbial replication. With the goal of eliciting a similar response, live attenuated versions of these intracellular microbes are being explored as vectors for cancer vaccines. *Listeria monocytogenes* (Lm) is a ubiquitous Gram-positive facultative intracellular bacterium typically found in soil and food that is nonpathogenic to immune competent individuals. A live-attenuated double deleted *Listeria* (LADD Lm) vaccine platform has been developed and tested in several early-stage clinical trials [17, 18]. The LADD vaccine strain has complete deletions of two virulence genes: *actA*, required for intracellular motility and cell-to-cell spread and internalin B (*inlB)*, required for direct hepatocyte invasion via the InlB-c-Met interaction [19]. The combination of the two deletions in the LADD platform results in a 1000x attenuation compared to WT Lm and limits liver toxicity by eliminating direct hepatocyte invasion and ActA-mediated cell-to-cell spread into hepatocytes from infected liver-resident Kupffer cells [20]. Importantly, the live-attenuated vaccine vector elicits a potent innate and adaptive immune response. Vaccine-induced inflammation also promotes APC maturation, antigen processing and presentation, and T cell expansion, resulting in a robust antigen-specific CD8^+^ T cell response [21]. In combination with our ability to construct *Listeria* that express tumor associated antigens, these inherent immunogenic properties make attenuated *Listeria* an attractive candidate for microbe-based cancer vaccines. Here, we describe the design of an EGFRvIII-expressing LADD Lm strain and demonstrate its efficacy in a preclinical tumor model. We demonstrate that this vaccine yields orders of magnitude higher EGFRvIII-specific CD8^+^ T cell responses compared to PepvIII-KLH *in vivo*, and effectively protects against EGFRvIII-expressing tumor challenge, indicating its potential for translation to treat EGFRvIII-expressing tumors in human trials.

## Materials and methods

### Antibodies, cells and reagents

Murine squamous cell carcinoma (SCCVII) cells [22] (generously provided by Walter T. Lee, Duke Cancer Institute, Durham NC) were grown in 10% RPMI-1640 with L-glutamine (ThermoFisher Scientific, Waltham, MA) supplemented with 10% heat-inactivated FBS (Atlas Biologicals, Fort Collins, CO), MEM Eagle Nonessential Amino Acid Solution, Penicillin-Streptomycin, L-glutamine, HEPES Buffer (Lonza, Basel, Switzerland), and Sodium Pyruvate Solution (ThermoFisher Scientific). Antibodies for flow cytometry include anti-mouse CD8α-PerCP-Cy5.5 (clone 5H10, ThermoFisher Scientific), CD4-FITC (clone RM4-5, eBioscience, San Diego, CA), CD154/CD40L-PE (clone MR1, eBioscience), anti-mouse IFNγ-APC (clone XMG1.2, eBioscience) and TNF-PE-Cy7 (clone MP6-XT22, BD Biosciences, Franklin Lanes, NJ). Peptides for restimulation were synthesized by A&A Labs (San Diego, CA). H-2K^k^ MHC-tetramers incorporating the defined EEKKGNYV peptide (EGFRvIII murine epitope) were obtained from the NIH Core Facility at Emory University, (Atlanta, GA).

### Animal models

Female C57BL/6, BALB/c, SJL and C3H/HeJ mice aged 5–8 weeks were obtained from Jackson Laboratories (Bar Harbor, ME) for use in these experiments. Animal protocols were approved by Providence Health & Services IACUC (Animal Welfare Assurance No. A3913-01).

### *L*. *monocytogenes* construction, growth and vaccination

All strains were based on the previously described parental LADD *Lm* (Δ*actA*Δ*inlB*) strain [17]. A 21-AA sequence (PASRALEEKKGNYVVTDHGSC) that overlaps the novel junction created by the 267-AA deletion in EGFRvIII was selected as the immunogenic peptide. Two EGFRvIII_20-40_-expressing constructs were designed, one with a single copy of the peptide and one with five copies. EGFRvIII_20-40_ was flanked by peptides predicted to facilitate cleavage of the construct by the immunoproteasome. The fusion protein included a C-terminal OVA_257-264_ (SIINFEKL) tag. The expression cassette was codon optimized for *Lm* and cloned downstream of an *actA* promoter in-frame with the 100 N-terminal amino acids of the *actA* gene. The expression cassette was cloned into a derivative of the pPL2 integration vector and stably integrated at the *tRNA*^Arg^ locus of the bacterial chromosome of vaccine platform strain as described previously [23]. To assess protein expression, vaccine strains were infected into the mouse dendritic cell line DC2.4 and cell lysates were harvested for western blotting using an antibody raised to the mature amino terminus of ActA as described [24]. To assess *in vivo* immunogenicity, BHI broth was inoculated with a single colony from a BHI agar plate and grown overnight at 37°C. Stationary phase cultures were split the next morning and allowed to return to midlog phase before dilution and immunization. All doses were confirmed by plating vaccination material. *Lm-EGFRvIII* or control *Lm-Ova* [25] were administered to mice at a dose of 1x10^5^-1x10^7^ CFU retro-orbital IV, depending on mouse strain. For comparison, groups of mice were vaccinated by subcutaneous injection of 50μg KLH-PEPvIII (generously provided by Celldex Therapeutics) along with 2μg murine recombinant GM-CSF (R&D Systems, Minneapolis, MN).

### Peptide stimulation, intracellular cytokine staining and flow cytometry

To enumerate EGFRvIII-specific T cells in following treatment, spleens were first dissociated using a 70μm cell strainer (ThermoFisher Scientific) and syringe. Red blood cells were lysed with ACK Lysing Buffer (Lonza) and the resultant splenocytes were washed three times in PBS, counted and diluted to 1x10^6^ viable cells/100μl. These cells were then stimulated with 1μg peptide and 1μl GolgiPlug (Becton Dickinson) for four hours, washed 3X with PBS and surfaced stained with anti-mouse CD8α-PerCP-Cy5.5 and CD4-FITC. Following the protocol for the BD Cytofix/Cytoperm Fixation/Permeabilization Solution Kit with GolgiPlug (Becton Dickinson), cells were then fixed, permeabilized and frozen at -80°C for future analysis. Upon thawing, cells were washed and stained with anti-mouse IFNγ-APC, TNF-PE-Cy7, and CD40L PE. Samples were analyzed using the BD LSRII flow cytometer (Becton Dickinson).

### T2 peptide binding assay

K^k^-expressing T2 cells (generously provided by Peter Cresswell, Yale University) are a Tap-deficient cell line that cannot assemble MHCI for presentation on the cell surface unless provided with exogenous MHC-binding peptides. The cells were incubated overnight with the indicated concentrations of peptide. Cells were washed, stained with an anti-K^k^ antibody (eBiosciences) and acquired using an LSR II flow cytometer.

### Plasmids and transfection

MSCV-XZ066-EGFRvIII was a gift from Alonzo Ross [26] (Addgene plasmid #20737) and pMSCV-loxp-dsRed-loxp-eGFP-Puro-WPRE was a gift from Hans Clevers [27] (Addgene plasmid #32702). SCCVII cells were transfected using Lipofectamine 2000 Transfection Reagent (Life Technologies, Carlsbad, CA, USA) according to the manufacturer protocol and selected in 2μg/ml puromycin as well as through three cycles of fluorescence-assisted cell sorting (FACS) for high endogenous GFP and RFP expression, respectively, to generate SCCVII-EGFRvIII and SCCVII-control cells, respectively.

### In vivo tumor assays

For tumor protection studies, female C3H mice were vaccinated with 1x10^5^ CFU of Lm-EGFRvIIIx5 or Lm-OVA at day -21 and again at day -7 relative to tumor challenge. At day 0, animals were injected subcutaneously with 2x10^6^ tumor cells (SCCVII-EGFRvIII or SCCVII-Vector) on the hind flank according to group. For dual flank experiments, female C3H mice were given simultaneous challenge of SCCVII-EGFRvIII and SCCVII-Vector on opposing hind flanks. Starting at day 7, tumor progression was monitored on both flanks with calipers to the endpoint of 12mm maximum diameter for either tumor, at which point the animal was euthanized. For long-term protection experiments, female C3H mice were vaccinated with 1x10^5^ CFU of *Lm-EGFRvIIIx5* or *Lm-Ova* at day -21 and again at day -7 relative to tumor challenge. At day 0, mice were injected subcutaneously with SCCVII-EGFRvIII or SCCVII-vector control on the hind flank according to group. For therapeutic studies, at d0 mice were injected subcutaneously with SCCVII-EGFRvIII as above and treated with *Lm-EGFRvIIIx5* or vehicle control on d3. Tumor size was monitored from day 7 with calipers to the endpoint of 12mm maximum diameter, at which point the animal was euthanized.

### Statistics

Data were analyzed and graphed using Prism (GraphPad Software, La Jolla, CA). Individual data sets were compared using Student’s T-test and analysis across multiple groups was performed using ANOVA with individual groups assessed using Tukey’s comparison. Overall survival of groups was compared using log rank test for differences in Kaplan-Meier survival curves.

## Results

### Construction of *Lm-EGFRvIII*

**Fig 1:**
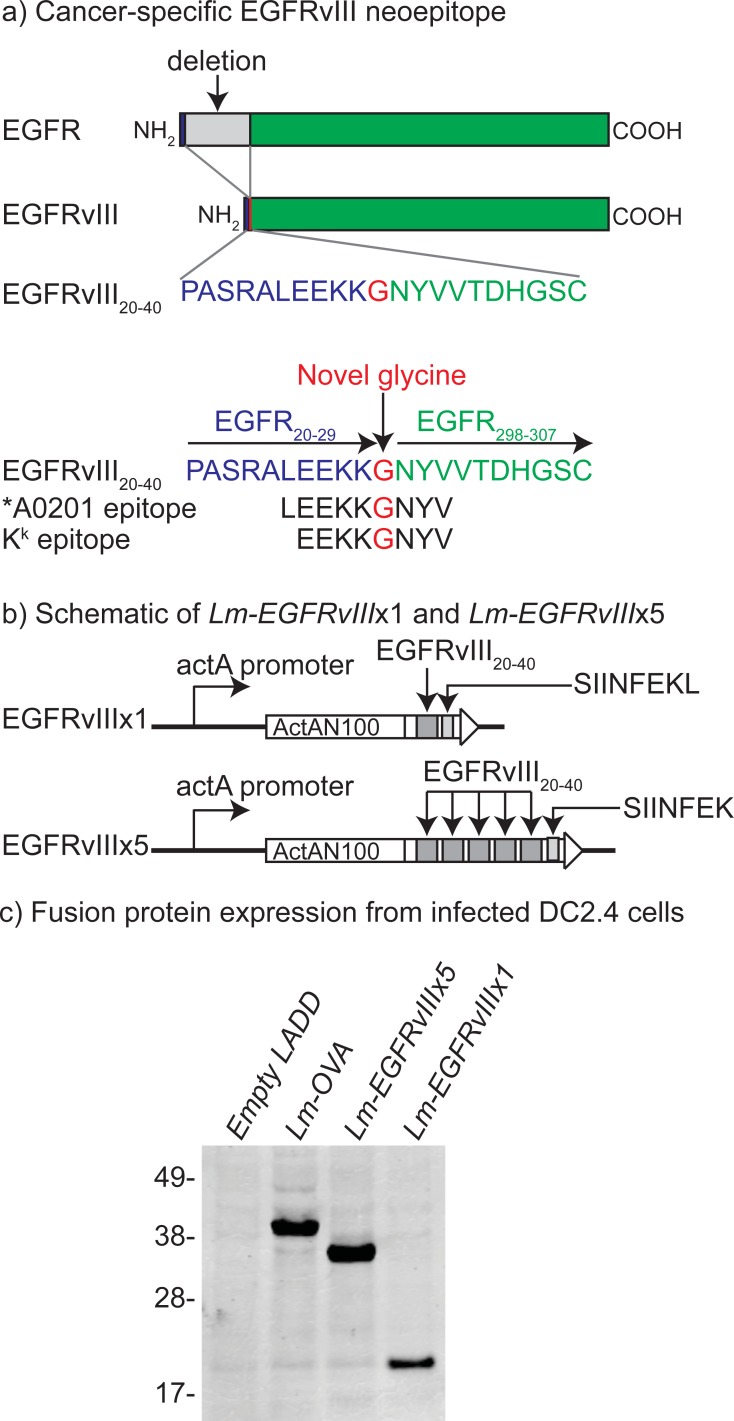
Construction and confirmation of Lm-EGFRvIII. a) Deletion of a 267 amino acid section of EGFR results in formation of EGFRvIII where amino acids from the amino terminus (blue) separated from the remainder of the EGFR molecule (green) by a novel glycine (red) at the junction. The novel EGFRvIII_20-40_ sequence incorporates a characterized human HLA A0201 binding neoepitope. b) One or 5 copies of EGFRvIII_20-40_, together with a single copy of the ovalbumin epitope SIINFEKL, were cloned in frame with the amino terminus of ActA and under control of the actA promoter in the parental LADD Lm vector. c) DC2.4s were infected with *Lm-EGFRvIII* x1, *Lm-EGFRvIII* x5 or controls and western blotted with antibodies specific for the mature amino terminus of the ActA fusion partner.

To generate an immunogenic EGFRvIII vaccine candidate, we cloned the EGFRvIII_20-40_ neoepitope formed as a result of deletion of a -267 amino acid section of EGFR, which results in a novel glycine at the deletion junction (**Fig 1A**). A larger, -21 amino acid peptide was selected (relative to the previously published 14-mer) to increase potential MHC class I binding epitopes. The initial EGFRvIII construct, EGFRvIII x1, contained a single copy of the EGFRvIII_20-40_ peptide flanked by peptides predicted to facilitate cleavage of the construct by the immunoproteasome. To investigate whether we could increase immunogenicity without also increasing pathogenicity, we developed an EGFRvIII x5 construct that contains five copies of the EGFRvIII_20-40_ peptide. Vaccine constructs were stably integrated each at the *tRNA*^*Arg*^ locus of the chromosome in the LADD *Listeria* strain (**Fig 1B**). Under control of the *actA* promoter, expression and secretion of the EGFRvIII fusion proteins will be maximal following escape of *Listeria* into the cytoplasm of the host cell. To assess the expression and secretion of full-length antigen fusion proteins, we routinely include the model epitope SIINFEKL (OVA_257-264_, from chicken ovalbumin) at the C-terminus. Additionally, the SIINFEKL-specific CD8^+^ T cell response serves as a standard to compare secretion and immunogenicity of different constructs *in vivo*. We first verified the secretion of the EGFRvIII constructs *in vitro*. To this end, we infected DC2.4 cells with *Lm-EGFRvIII* x1, *Lm-EGFRvIII* x5 or controls and prepared cytoplasmic extracts 7 hours post infection. All of the EGFRvIII_20-40_ containing constructs were detected indicating effective uptake and protein secretion in antigen presenting cells compared to controls (**Fig 1C**).

**Fig 2:**
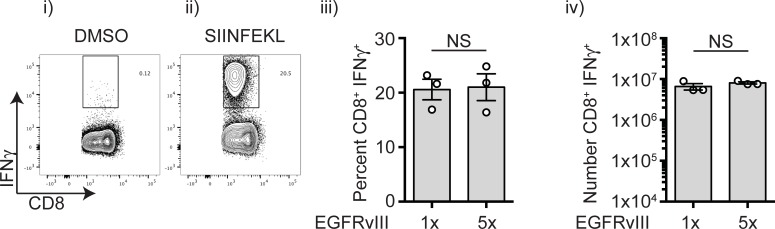
In vivo vaccination with *Lm-EGFRvIII* results in antigen specific T cell responses. C57BL/6 mice were treated with *Lm-EGFRvIII* x1 and *Lm-EGFRvIII* x5 and seven days later splenocytes were tested for their response to i) DMSO vehicle, or ii) SIINFEKL peptide by intracellular cytokine staining. iii) Percent of CD3+CD8+ T cells and iv) absolute number of SIINFEKL-specific splenocytes. Each symbol represents one animal. NS = not significant. (Student’s T-test).

A reliable metric of antigen secretion efficiency is to include the K^b^-restricted SIINFEKL peptide at the C-terminus of a construct, and then assess the magnitude of the SIINFEKL-specific CD8+ T cell response in vivo. C57BL/6 mice were immunized with 1x10^7^ colony forming units (CFU) of either Lm-EGFRvIII x1 or Lm-EGFRvIII x5, each containing a single copy of the SIINFEKL peptide. Seven days later, spleens were harvested and the number and frequency of SIINFEKL-specific CD8^+^ T cells was determined by IFN-γ intracellular staining (ICS) in response to peptide stimulation. We found that following a single immunization approximately 20% of the CD8^+^ T cells in the spleen were SIINFEKL specific, and the SIINFEKL-specific responses elicited by the Lm-EGFRvIII x1 and Lm-EGFRvIII x5 strains were equivalent (**Fig 2**). These data demonstrate that the construct promoted efficient and comparable secretion of the antigenic polypeptides from the bacteria into the infected cell cytoplasm, resulting in robust antigen-specific CD8+ T cell responses following immunization.

### Identification of a class I restricted EGFRvIII epitope

**Fig 3:**
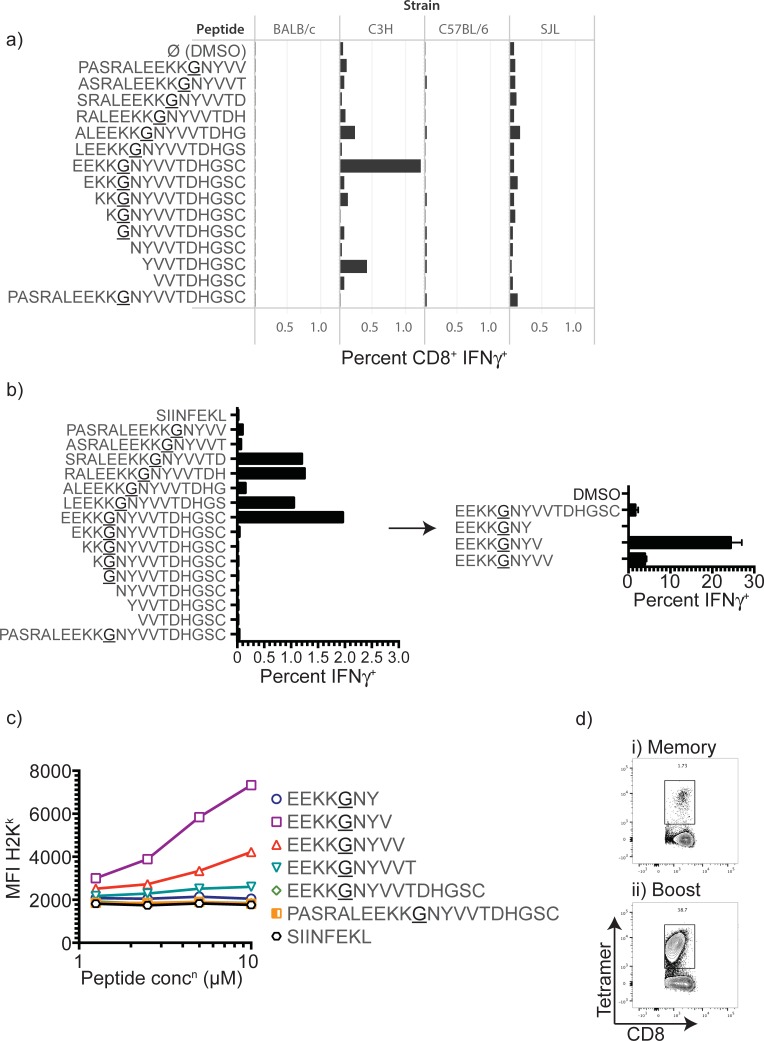
Identification of a novel murine class I epitope from EGFRvIII. a) BALB/c, C57BL/6, C3H and SJL mice were vaccinated with Lm-EGFRvIII and 7 days later tested by ICS for EGFRvIII-specific T cells using a library of overlapping peptides from EGFRvIII_20-40_, or a DMSO vehicle control. Spleens from three mice per strain were used for restimulation. The percentage of IFN-γ+ events within the CD3+CD8+ T cell gate are shown for each peptide; b) Splenocytes from C3H mice primed and boosted with Lm-EGFRvIII were stimulated using a library of overlapping peptides from EGFRvIII_20-40_, or SIINFEKL as a negative control. A subgroup of peptides within the previously identified 15-mer were used to define the optimal 9-mer peptide by ICS. c) H2K^k^ binding of EGFRvIII or control peptides was assessed by stabilization of surface MHC I using flow cytometry. d) Identification of EGFRvIII-specific T cells in the spleen following prime-boost vaccination with *Lm-EGFRvIII* using H2K^k^-tetramers folded with the defined peptide EEKKGNYV.

Thus far, murine class I-restricted epitopes in EGFRvIII have not been defined, which has limited the ability to comparatively evaluate vaccination approaches. In addition, using the precise class I binding epitope improves responsiveness in restimulation assays (ICS or ELISpot) and allows assembly of MHC-peptide tetramers. To identify murine class I-restricted epitopes in EGFRvIII, as well as to perform a preliminary evaluation of immunogenicity, we immunized C57BL/6 (H-2^b^), BALB/c (H-2^d^), C3H/HeJ (H-2^k^) and SJL (H-2^s^) mice with 1x10^7^ CFU of each EGFRvIII_20-40_ expressing strain and seven days later determined the frequency and specificity of EGFRvIII-specific CD8^+^ T cells in the spleen by IFN-γ ICS. Specificity of the response was determined using an EGFRvIII_20-40_ overlapping peptide library comprised of 15-amino acid peptides overlapping by 14 amino acids. We found that the greatest peptide-specific CD8^+^ T cell response to EGFRvIII occurred in the C3H strain (H-2^k^) (**Fig 3A**). In order to confirm and refine the exact amino acid sequence of the EGFRvIII class I-binding peptide, a cohort of C3H mice were primed with 1x10^7^ CFU Lm-EGFRvIII x5 and spleens were either harvested seven days later or boosted 21 days later with 1x10^5^ CFU Lm-EGFRvIII x5 and the CD8^+^ T cell response was screened with the same peptide library. Additionally, we included 7-, 8- and 9-mer peptides refined from the 15-mer identified in the primary immunogenicity assay. CD8^+^ T cells isolated from the spleens of primed and boosted mice responded to several of the 15-amino acid peptides (**Fig 3B**). Using 7-, 8- and 9-mers from this sequence in the primary assay, we refined this peptide to identify the 8-amino acid peptide EEKKGNYV as a novel class I restricted epitope (**Fig 3B**). The identified 8-mer, EEKKGNYV, was predicted to bind K^k^ according to available prediction algorithms (SYFPEITHI). To confirm binding, we used a K^k^-expressing T2 cell line, where peptide binding stabilizes the MHC class I molecule (in this case, K^k^) on the cell surface. When MHC K^k^ expression was evaluated with increasing concentrations of the peptide, EEKKGNYV (EGFRvIII_26-33_) was confirmed as the optimal binder of K^k^ (**Fig 3C**). Finally, K^k^-EGFRvIII_26-33_ tetramers were able to specifically identify T cells expanded following vaccination of C3H mice with Lm-EGFRvIII (**Fig 3D**). These results define a novel K^k^-restricted epitope in EGFRvIII that can be used to assess the potency of EGFRvIII-containing vaccine candidates.

### Immunogenicity of EGFRvIII-expressing vaccine candidates

**Fig 4:**
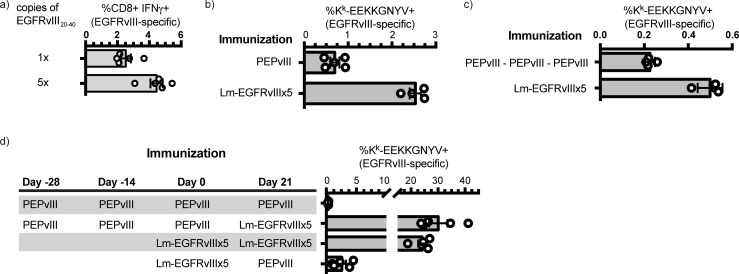
Immunogenicity of EGFRvIII vaccine candidates. a) EGFRvIII_26-33_ -specific CD8+ T cell responses in C3H mice vaccinated with Lm-EGFRvIIIx1 or Lm-EGFRvIIIx5, determined by IFN-γ ICS on day 7. b) Primary immunogenicity of PEPvIII and Lm-EGFRvIIIx5 in C3H mice, represented as the frequency of K^k^-EGFRvIII_26-33_ -tetramer+ cells within the CD3+CD8+ T cell population. c) 21 days after immunization with Lm-EGFRvIIIx5 or a 3 dose regimen of PEPvIII, the frequency of K^k^-EGFRvIII_26-33_ -tetramer+ cells within the CD3+CD8+ T cell population was determined. d) C3H mice were vaccinated x3 with PEPvIII, or a single dose of Lm-EGFRvIIIx5 as indicated. 21 days after the last vaccination, mice were boosted with PEPvIII or Lm-EGFRvIIIx5. Five days later, the frequency of K^k^-EGFRvIII_26-33_ -tetramer+ cells within the CD3+CD8+ T cell population was determined. Each symbol represents one animal. Bars represent mean ± SEM of groups containing 4–5 animals. Each figure represents a single experiment from a minimum of two replicates. (ANOVA).

A high frequency of tumor-specific MHC-peptide complexes on dendritic cells following vaccination is important for maximizing TCR signaling. This was the rationale for engineering the Lm-EGFRvIIIx5 candidate, though it remained unclear if this would result in improved T cell responses to a complex microbial-based vaccine. First, we compared the primary EGFRvIII-specific CD8^+^ T cell response after immunization with the EGFRvIII x1 or x5 strain. Female C3H mice were immunized with 1x10^5^ CFU of each strain, and seven days later spleens were harvested and the frequency of EGFRvIII_26-33_–specific CD8^+^ T cells was determined by ICS. Consistent with our hypothesis, the inclusion of multiple copies of EGFRvIII_20-40_ elicited more EGFRvIII-specific CD8^+^ T cells than the EGFRvIII x1 single copy variant (**Fig 4A**). Together with the data (**Fig 2**) showing equivalent transcription, translation and secretion of the 1x and 5x polypeptides (both strains have only a single copy of SIINFEKL), these findings demonstrate that increasing the frequency of class I-binding peptides directly impacts the magnitude of the primary CD8+ T cell response.

To compare the immunogenicity of the LADD *Lm* vaccine platform compared to the existing peptide vaccine, mice were vaccinated with *Lm-EGFRvIII x5* or the EGFRvIII-specific 14-mer peptide coupled to keyhole limpet hemocyanin and co-administered with GM-CSF (referred to as PEPvIII in subsequent text and figures) (**Fig 4B–4D**). First, we assessed T cell responses 7 days after a single vaccination with Lm-EGFRvIIIx5 or PEPvIII, and found that Lm-EGFRvIIIx5 consistently elicited 3–5 fold more EGFRvIII_26-33_ -specific CD8+ T cells than PEPvIII (**Fig 4B**). Next, we compared a dosing regimen closer to what has been used in the clinic with rindopepimut; i.e., multiple immunizations separated by 14 days. Mice were immunized with PEPvIII on days -28, -14, and 0, or with a single dose of Lm-EGFRvIIIx5 on day 0. 21 days later, the frequency of EGFRvIII_26-33_ -specific CD8+ T cells was determined using K^k^-EEKKGNYV tetramers (**Fig 4C**). Consistent with the primary CD8+ T cell response, the frequency of EGFRvIII-specific CD8+ T cells was greater following Lm-EGFRvIIIx5 immunization than PEPvIII. On day 21, separate cohorts were boosted with either PEPvIII or Lm-EGFRvIIIx5 as either a homologous or heterologous prime-boost. Five days later, the frequency of EGFRvIII_26-33_ -specific CD8+ T cells in the spleen was determined. Mice boosted with Lm-EGFRvIIIx5, whether they were primed with PEPvIII or Lm-EGFRvIIIx5, demonstrated robust secondary expansion (**Fig 4D**). Conversely, mice boosted with PEPvIII realized comparatively modest secondary expansion. These findings are consistent with our understanding of inflammation as a driver of CD8+ T cell expansion. Together, these data demonstrate that a prime-boost of *Lm-EGFRvIII x5* is significantly more effective at generating EGFRvIII-specific CD8+ T cells than a prime-boost regimen of PEPvIII (**Fig 4D**). In addition, *Lm-EGFRvIII x5* is effective at boosting responses primed with PEPvIII, suggesting that it could be applied in patients who have only weak responses to rindopepimut, to boost their EGFRvIII-specific immunity. These data demonstrate that a multicopy EGFRvIII_20-40_-expressing LADD *Lm*-based vaccine readily promotes expansion of antigen-experienced CD8^+^ T cells and can be repeatedly administered to generate long-term antigen-specific immunity.

### Evaluation of *Lm-EGFRvIII* as an anti-cancer therapeutic

**Fig 5:**
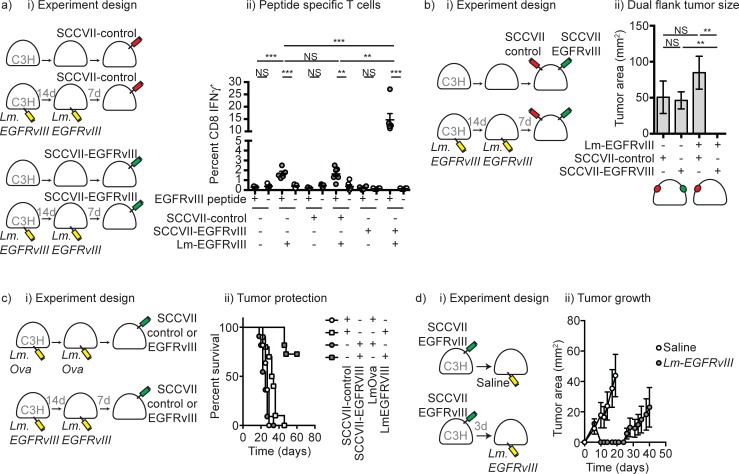
Control of EGFRvIII-expressing tumors by Lm-EGFRvIII. a) C3H mice were left untreated or vaccinated with Lm-EGFRvIIIx5 twice separated by 14 days. 7 days following the last vaccine, mice were challenged with SCCVII-control or SCCVII-EGFRvIII. Ii) 7 days following tumor challenge, EGFRvIII-specific CD8 T cells in the spleen were quantified by IFN-γ ICS. b) i) C3H mice were left untreated or vaccinated with Lm-EGFRvIII twice separated by 14 days. 7 days following the last vaccine, mice were challenged with SCCVII-control on one flank and SCCVII-EGFRvIII on the opposite flank. ii) Size of tumors in unvaccinated animals (left two columns) or vaccinated animals (right two columns) d10 following tumor challenge. c) C3H mice were vaccinated with Lm-OVA as a vector control or Lm-EGFRvIII twice separated by 14 days. 7 days following the last vaccine, mice were challenged with SCCVII-control or SCCVII-EGFRvIII and followed for ii) survival of vaccinated animals. d) C3H mice were implanted with SCCVII-EGFRvIII and left untreated or vaccinated with a single dose of Lm-EGFRvIII on d3 following tumor challenge. Graphs show average tumor growth of treatment groups. Key: * = p<0.05; ** = p< 0.01; *** = p<0.001; **** = p<0.0001 (a- ANOVA; b- T-test; c,d Log rank).

To test this agent as an anti-cancer therapeutic, we transfected the squamous cell carcinoma cell line SCCVII that is syngeneic to C3H mice with an EGFRvIII plasmid construct (SCCVII-EGFRvIII) or the plasmid construct backbone alone (SCCVII-control) and generated stable cell lines. C3H mice were primed with Lm-EGFRvIIIx5 or vehicle control and 14 days later rechallenged with Lm-EGFRvIIIx5 or vehicle control. 7 days later mice were implanted with SCCVII-EGFRvIII or with SCCVII-control (**Fig 5AI**). 7 days following tumor implantation, mice were evaluated for EGFRvIII-specific T cell responses. CD8^+^ T cell responses to EGFRvIII were not detectable in unvaccinated mice and had declined in mice receiving no further treatment or implanted with SCCVIII-control tumors (**Fig 5AII**). However, implantation of SCCVII expressing EGFRvIII significantly boosted the EGFRvIII-specific CD8^+^ T cell responses (**Fig 5AII**), indicating that the T cells generated by vaccination further expanded following recognition of antigens present in the cancer cells. To determine whether this resulted in antigen-specific tumor control, C3H mice were primed with Lm-EGFRvIIIx5 or vehicle control and 14 days later boosted with Lm-EGFRvIIIx5 or vehicle control. 7 days following the boost vaccine mice were implanted with SCCVII-EGFRvIII on one flank and SCCVII-control on the opposite flank (**Fig 5BI**). Mice given only vehicle developed tumors on both flanks, while mice vaccinated with Lm-EGFRvIII only developed SCCVII-control tumors and failed to develop EGFRvIII expressing SCCVII tumors (**Fig 5BII**). The progressive growth of the antigen-negative tumor prevented long-term monitoring of these animals to evaluate late outgrowth of tumors due to loss of antigen-specific control. Therefore, we evaluated protection against growth of a single tumor implanted in each animal. To evaluate long-term protection against tumor outgrowth, C3H mice were primed with Lm-EGFRvIIIx5 or Lm-OVA as an irrelevant antigen control, and 14 days later boosted with Lm-EGFRvIIIx5 or Lm-OVA. 7 days following the boost vaccine mice were implanted with either SCCVII-EGFRvIII or SCCVII-control. Mice vaccinated with Lm-EGFRvIIIx5 exhibited long term protection against growth of tumors expressing EGFRvIII. These data demonstrate Lm-EGFRvIIIx5 is an effective vaccine that results in antigen-specific protection against EGFRvIII-expressing SCCVII tumors (**Fig 5C**). To determine whether the vaccine was also effective in a therapeutic setting, mice bearing SCCVII-EGFRvIII were left untreated or vaccinated with *Lm-EGFRvIIIx5* and followed for outcome. Antigen-specific vaccination resulted in a significant extension in survival (p<0.01) with 13/15 mice cured of their tumor across experimental repeats (**Fig 5D**). These data demonstrate that in the preclinical setting, *Lm-EGFRvIIIx5* is an effective anti-cancer therapeutic for EGFRvIII-expressing tumors.

## Discussion

We demonstrate that potent EGFRvIII-specific CD8^+^ T cell responses can be elicited using LADD, an attenuated *L*. *monocytogenes* strain, as the vaccine vector. Using this approach we were able to define an EGFRvIII-specific CD8^+^ T cell epitope in C3H mice and demonstrate control of EGFRvIII-expressing tumors in immunocompetent mice. LADD Lm-based vectors are not susceptible to the same vector-specific neutralizing immunity that limits viral vaccine vectors [28] and this represents a novel potent candidate vaccine for patients with EGFRvIII-expressing tumors.

Previous reports using EGFRvIII-targeted vaccines have failed to demonstrate an EGFRvIII-specific T cell response in mice that could be measured by ICS or ELISpot [29–31]. When compared with results from previous pre-clinical studies using recombinant protein or peptide-pulsed dendritic cells, we observe a significantly more potent *in vivo* T cell response to our EGFRvIII-expressing vaccine. In addition to using a potent, live microbial vaccine vector, we identified a novel K^k^-restricted epitope, EEKKGNYV, within the EGFRvIII_20-40_ immunogen. Using this defined class I-restricted peptide, we were able to determine the magnitude and quality of the EGFRvIII-specific CD8^+^ T cell response to *Lm-EGFRvIII* and the previously described PepvIII-KLH conjugate. In addition, we were able to test the efficacy of the vaccine candidate using a squamous cell carcinoma cell line, engineered to express full-length EGFRvIII, which was syngeneic to the C3H mouse strain and therefore able to present the EEKKGNYV peptide on H2^k^. This direct presentation was not possible on prior tumor models that were tested in mice expressing MHC class I H2^d^ haplotypes [29] or in human cells in immunosuppressed animals [30] and in these models tumor control was associated with antibody rather than T cell responses [31].

Targeting T cell responses to EGFRvIII rather than antibody responses is particularly relevant in view the failure of the phase III PepvIII-KLH conjugate trial [16] and the promising results of a recent phase 1 trial infusing EGFRvIII-specific chimeric antigen receptor (CAR) T cells into recurrent glioblastoma patients. In this latter study, autologous T cells were engineered to express an EGFRvIII-binding CAR signaling through CD3ζ and the 4-1BB costimulation domain and then infused back into patients. These T cells were observed to effectively traffic to tumors and significantly decreased EGFRvIII expression [32]. In patients, the PepvIII-KLH conjugate did generate a detectable antibody response to EGFRvIII, and the clinical data suggested that antibody responses correlated with clinical activity [16]. We demonstrate that PEPvIII does generate an antigen-specific T cell response to the neoantigen within EGFRvIII in C3H mice, and this vaccine had been shown to generate CD8 T cell-mediated tumor control in murine models [31]. While one of the strengths of the *Listeria* platform is that repeated vaccination is not limited by neutralizing antibody responses, antibody responses may be elicited following *Listeria* infection and contribute to T cell immunity [33]. Further studies are necessary to determine whether CAR T cell transfer or endogenous vaccination yields superior results in clinical settings, and whether antibodies contribute to tumor control by *Lm-EGFRvIII*.

EGFRvIII-expressing cancer cells frequently represent a subclonal population of the tumor, and as discussed above, in the clinical studies tumor expression of EGFRvIII was lost equivalently in both the rindopepimut and control-treated patients [16]. While this suggests that the cancer cells have a potential path to immune escape, the EGFRvIII+ cancer cells are more resistant to chemotherapeutic agents such as paclitaxel and cisplatin [9] and a subclonal population of EGFRvIII+ cells can promote survival of EGFR wt neighbors [11]. Therefore, while successful clearance of EGFRvIII+ cancer cells would not be expected to affect EGFR wt neighbors, it has the potential to render the tumor significantly more susceptible to conventional therapies as part of combination treatments. Moreover, a strong tumor-specific T cell response has the potential to dramatically change the inflammatory environment of the tumor, and support epitope spreading to common mutations shared between EGFRvIII+ and EGFR wt neighbors. Thus the selectivity and specificity of the EGFRvIII epitope has the potential to immunoedit the tumor to a more treatable state, and provide a focus for further immune control of residual disease.

Given our experience constructing and producing *L*. *monocytogenes*-based vaccines, data with this vector in previous phase I clinical trials, and the demonstrated immunogenicity of EGFRvIII_20-40_-expressing LADD Lm, we believe Lm-EGFRvIII to be an ideal candidate for testing in patients with EGFRvIII-expressing cancers. The studies will include validation of the EGFRvIII neo-eptiope as a target for CD8 T cells in patients. As such, a phase 1 study testing ADU-623, a *Listeria* vaccine of similar construction expressing EGFRvIII-NY-ESO-1, is currently ongoing in patients with recurrent grade 3/4 glioblastomas (ClinicalTrials.gov identifier: NCT01967758).
